# Recent progress on nanotechnologies for enhancing blood‐brain barrier permeability

**DOI:** 10.1002/smo.20240052

**Published:** 2025-03-20

**Authors:** Qibin Liu, Zhuoqian Chen, Anthony Guiseppi‐Elie, Fanling Meng, Liang Luo

**Affiliations:** ^1^ National Engineering Research Center for Nanomedicine College of Life Science and Technology Huazhong University of Science and Technology Wuhan China; ^2^ Wuhan Pulmonary Hospital Wuhan Institute for Tuberculosis Control Wuhan China; ^3^ Key Laboratory of Molecular Biophysics of the Ministry of Education College of Life Science and Technology Huazhong University of Science and Technology Wuhan China; ^4^ Bioelectronics, Biosensors and Biochips (C3B®) Department of Biomedical Engineering Texas A&M University College Station TX USA; ^5^ Hubei Key Laboratory of Bioinorganic Chemistry and Materia Medica School of Chemistry and Chemical Engineering Huazhong University of Science and Technology Wuhan China

**Keywords:** biological activity, imaging agents, nanoparticles, nanotechnology, polymers

## Abstract

The blood‐brain barrier (BBB) is a substantial impediment to effectively delivering central nervous system (CNS) therapies. In this review, we provide a comprehensive dissection of the BBB's elaborate structure and function and discuss the inherent limitations of conventional drug delivery mechanisms due to its impermeability. We summarized the creative deployment of nanocarriers, the astute modification of small molecules to bolster their CNS penetration capabilities as well as the burgeoning potential of magnetic nanoparticles and optical techniques that are positioned to enable more precise and targeted drug delivery across the BBB and we discuss the current clinical application of some nanomedicines. In addition, we emphasize the indispensable role of artificial intelligence in designing novel materials and the paramount significance of interdisciplinary research in surmounting clinical challenges associated with BBB penetration. Our review meticulously integrates these insights to accentuate the impact of nanotechnological innovations in BBB research and CNS disease management. It presents a promising trajectory for the evolution of patient care in neurological disorders and suggests that these scientific strides could lead to more efficacious treatments and improved outcomes for those afflicted with such conditions.

## Graphical abstract

In this review, we summarize innovative strategies for harnessing the power of nanotechnology to enhance blood‐brain barrier permeability, a key challenge for central nervous system (CNS) drug delivery. We overview structural innovation of nanocarriers, improvements in targeting strategies, and nanotechnology‐enhanced brain imaging. This review will open new avenues for developing more effective treatments for CNS diseases.

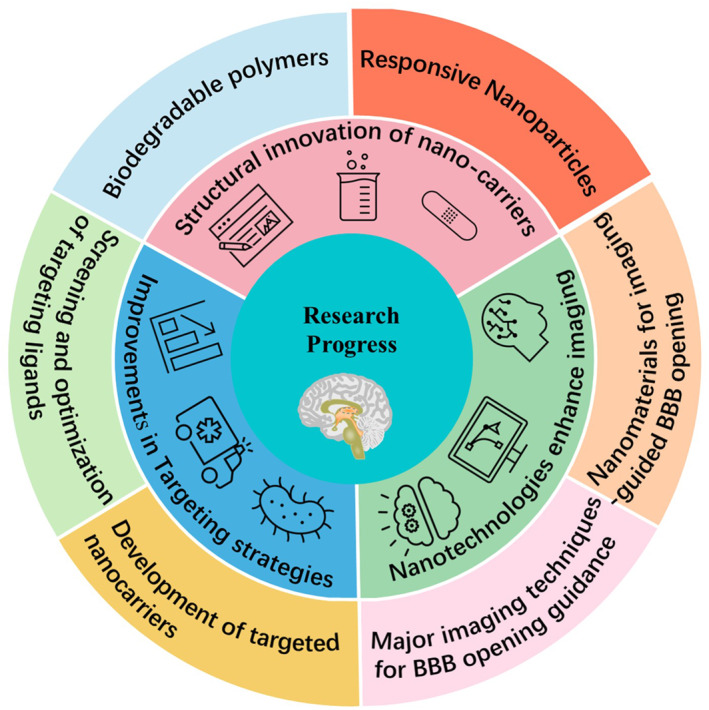

## 1 INTRODUCTION

**FIGURE 1:**
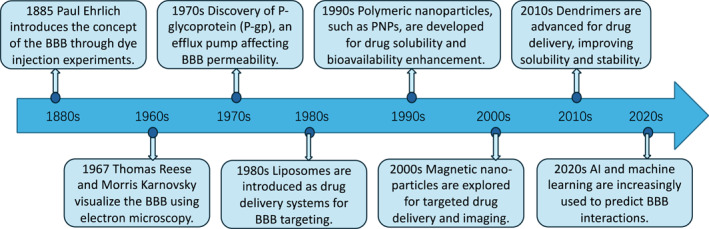
A timeline of significant milestones in BBB‐related nanotechnology research. BBB, blood‐brain barrier.

The blood‐brain barrier (BBB) serves as a vital protective barrier that safeguards the central nervous system (CNS) from harmful substances such as toxins and pathogens that infiltrate and circulate in the bloodstream.^[1]^ The concept of the BBB was first introduced in the pioneering work of the German physician Paul Ehrlich in 1885.^[2]^ He observed that when he injected a dye into the bloodstream of a mouse, the dye diffused throughout the body's tissues but was notably excluded from the brain and spinal cord.[^3^, ^4^] This intriguing phenomenon laid the foundation for the understanding of the BBB. As shown in Figure 1, Further advancements in the field were made in 1967 when Thomas Reese and Morris Karnovsky utilized electron microscopy to visualize the BBB,^[5]^ thereby elucidating the critical role played by endothelial cells in its form and function.^[6]^ This discovery significantly advanced the understanding of the BBB’s structural and functional properties, highlighting its crucial role in maintaining the integrity of the CNS.

**FIGURE 2:**
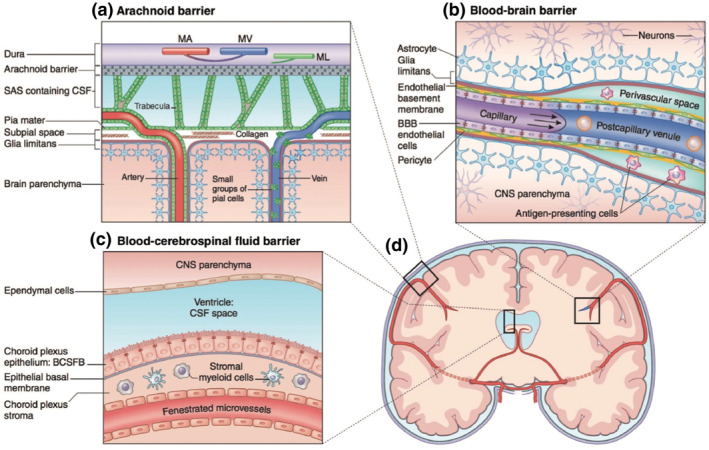
Barriers of the CNS. The three main barriers of the CNS include (a) the arachnoid barrier, (b) the blood–brain barrier, and (c) the blood–cerebro‐spinal‐fluid barrier, all of which are identifiable in (d) a schematic coronal brain section. Of the three barriers, the BBB maintains the closest proximity to the brain parenchyma, and therefore offers the most viable opportunity for CNS drug delivery.^[7]^ BBB, blood‐brain barrier; CNS, central nervous system. Reproduced with permission. Copyright 2018, Wiley‐Blackwell.

The BBB is a highly selective semipermeable membrane that strategically separates the blood from the brain's interstitial fluid,^[7]^ as shown in Figure 2. It is endowed with unique characteristics that actively and passively regulate the exchange of substances, effectively preventing the penetration of harmful substances into the cerebrospinal fluid.^[8]^ This barrier is intricately formed by the cerebral microvascular system, which comprises highly specialized endothelial cells. These cells are enveloped by pericytes and a robust basement membrane and are further supported by the terminal processes of astrocytes, which contribute significantly to the barrier's structural and metabolic functions.[^9^, ^10^] The BBB is thus both a cellularized physical structure and a physiological concept.

The BBB's impermeability to most therapeutic compounds^[11]^ presents a formidable challenge in modern medicine as it restricts the delivery of essential drugs to the brain. The barrier’s function is modulated by a complex interplay of perivascular cells, including astrocytes, pericytes, microglia, and neurons. These cells form the neurovascular unit, a specialized structure characterized by polarized endothelial cells with high electrical resistance.^[12]^ This intricate network ensures the BBB's role as a vigilant guardian of the CNS, maintaining a delicate balance between protection and the need for therapeutic access.

The first experiment in enhancing the BBB permeability involved the use of microbubbles as a cavitation agent, and the experimental results demonstrated a fourfold increase in the BBB cellular model outcomes.^[13]^ These pioneering experiments, aimed at augmenting the permeability of the BBB, leveraged the mechanical forces of expanding microbubbles as a cavitation agent. Subsequent studies have focused on manipulating the apparent permeability of the BBB by altering the perm‐selective properties of drug compounds. The goal is to assess the drug's ability to traverse the BBB epithelium, thereby gaining insights into its potential for therapeutic applications. Through meticulous observation, researchers can detect variations in the apparent permeability of the test compound either in the epithelial or basal directions.^[14]^ This methodological refinement allows for a more nuanced understanding of how different compounds interact with the BBB, paving the way for the development of more effective strategies in CNS drug delivery.

**FIGURE 3:**
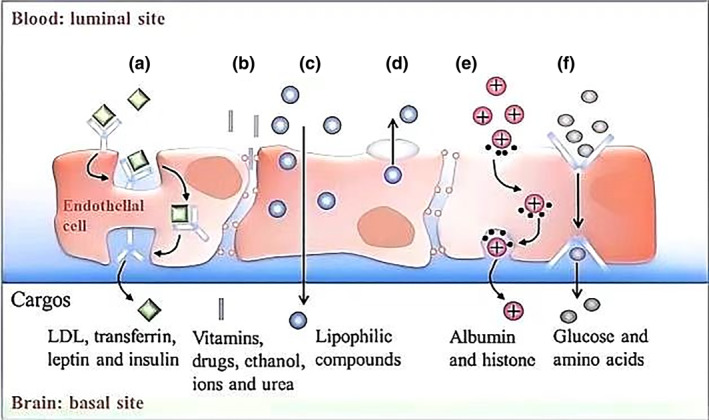
Transport processes at the capillary endothelium of BBB. (a) Receptor‐mediated transcytosis; (b) paracellular diffusion; (c) transcellular diffusion; (d) efflux pumps; (e) adsorptive trans‐ cytosis; (f) transporter‐mediated transcytosis. BBB, blood‐brain barrier; LDL, low‐density lipoproteins.^[18]^ Reproduced with permission. Copyright 2021, Science Press.

The BBB is distinguished by its highly restrictive permeability, a feature crucial for maintaining the integrity of the CNS. This selectivity is typically assessed by measuring the trans endothelial electrical resistance (TEER) in BBB models, which provides an indication of the barrier's tightness, and by calculating the permeability coefficients of tracer molecules across or between endothelial cells.[^15^, ^16^] The BBB’s impermeability is further underscored by its characteristic tight junctions and the scarcity of vesicles within the endothelial cells lining the cerebral arteries, capillaries, and veins.^[17]^ These structural elements collectively minimize the non‐selective entry of molecules into the brain.^[18]^ Moreover, the BBB plays a pivotal role in CNS homeostasis by facilitating the selective transport of essential ions, nutrients, proteins, and waste products between the blood and the brain. The unique endothelial cells of the BBB are equipped with specialized transport mechanisms that ensure a balanced exchange,^[19]^ thereby supporting the physiological needs of the brain while protecting it from potentially harmful substances,[^20^, ^21^] as shown in Figure 3. This dual function underscores the BBB’s critical role in safeguarding the brain's microenvironment and underscores the importance of understanding its mechanisms for developing effective therapeutic strategies.

Particle size is a critical factor influencing the interaction of nanomaterials with the BBB. Nanoparticles with a diameter smaller than 200 nm are generally preferred for BBB penetration due to their ability to evade reticuloendothelial system clearance and enhance permeability across endothelial cells.^[22]^ Smaller particles can also diffuse more easily through the tight junctions and fenestrations present in the BBB. However, particles that are too small may suffer from rapid renal clearance, thus optimizing particle size for both BBB penetration and systemic circulation is essential.

The surface charge of nanoparticles plays a significant role in their interaction with the BBB.^[23]^ Nanoparticles with a neutral or slightly negative zeta potential are less likely to interact non‐specifically with the negatively charged glycocalyx layer of the endothelial cells, potentially enhancing their passage across the BBB.

The shape of nanomaterials plays a critical role in their ability to cross the BBB.[^24^, ^25^] Spherical nanoparticles, being dimensionally isotropic and of a consistent contact area for cellular receptors, tend to interact uniformly with the endothelial cells of the BBB and are preferentially taken up via endocytosis. Rod‐shaped nanoparticles present a higher surface to volume (S/V) in their interactions with the membrane. Rods may preferentially align along the basal‐luminal axis of endothelial cells, facilitating certain transport mechanisms, but this alignment might also increase resistance to uptake compared to spheres. Disk‐shaped nanoparticles may exhibit distinct adhesion and interaction behaviors due to their flat surfaces, which can enhance or hinder uptake depending on their orientation relative to the cell surface. Other exotic shapes (e.g., cubes, stars) introduce unique surface interactions, with sharp edges or protrusions potentially enhancing adhesion or triggering specific cellular pathways. Shape also influences particle hydrodynamics and margination with spheres showing more uniform behavior compared to rods and disks. These different shapes may preferentially activate distinct endocytosis pathways (e.g., clathrin‐mediated [spheres], caveolae‐mediated [rods], or macropinocytosis). Finally, shape determines the effective surface area for ligand functionalization with S/V ratios being $\frac{3}{r}$ (for a sphere of radius, *r*), $\frac{2}{rL}+\frac{2}{rL^{2}}$ (for a rod of radius, *r* and length, *L*) and $\frac{2}{r}+\frac{1}{tr^{2}}$ (for a disc of radius, *r* and thickness, *t*) and so may influence BBB transport via enhanced ligand density of targeting molecules.^[26]^ Ligand functionalization of nanoparticles allows for active targeting of specific receptors overexpressed on the BBB, such as transferrin receptors and insulin receptors.^[27]^ This targeted approach can significantly enhance the selectivity and uptake of nanoparticles by the brain. The choice of ligand is crucial as it must have high affinity and specificity for the target receptor to ensure efficient targeting.

Understanding and modulating these physicochemical properties are essential for the design of effective nanomaterials for BBB penetration. Particle size, shape, surface charge, and ligand functionalization are interrelated properties that must be finely balanced to achieve optimal BBB permeability and therapeutic efficacy. Further research is needed to elucidate the complex interactions between these properties and their impact on the pharmacokinetics and pharmacodynamics of nanomaterials in the CNS.

**FIGURE 4:**
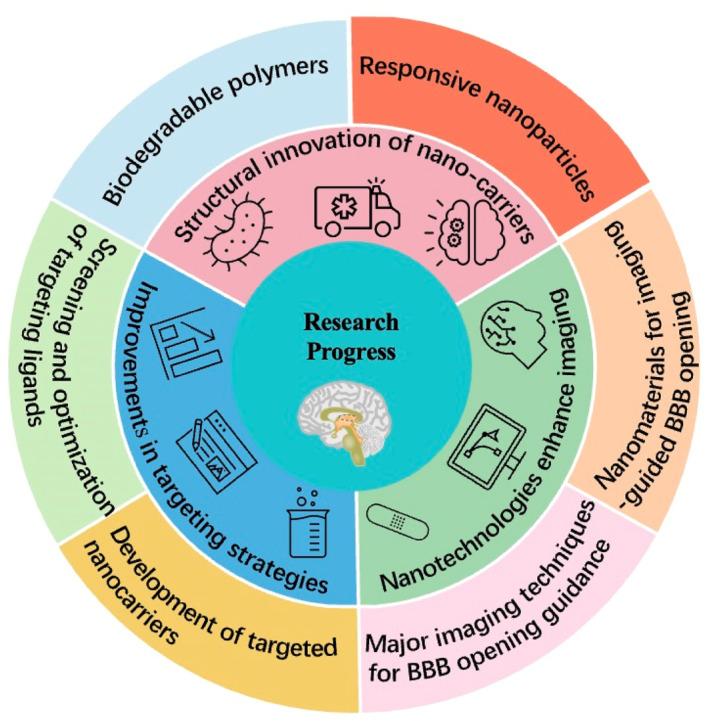
Schematic illustration of recent advancements in nanotechnology for enhancing BBB permeability that will be discussed covering innovations in nano‐carrier structures, targeting strategies, imaging‐guided BBB opening techniques, and clinical complexities. It also explores future research directions, including the role of AI in material design and interdisciplinary collaboration, concluding with the impact of these developments on BBB research and CNS disease treatment. BBB, blood‐brain barrier; CNS, central nervous system.

Nanotechnology has emerged as a promising platform offering innovative solutions to the longstanding challenge of delivering drugs to the CNS and treating associated disorders.^[25]^ This cutting‐edge field holds the potential to revolutionize therapeutic approaches, moving beyond conventional methods that have often proven unsatisfactory.^[28]^ Specifically, nanocarriers can be meticulously engineered to deliver a specific therapeutic payload to targeted tissues, thereby enhancing the precision and efficacy of treatments.^[29]^ Advancements in nanotechnology and biomaterials are reshaping the landscape of neurological disorder treatment.^[30]^ Nanomaterials, with their capacity to navigate the BBB and enable targeted drug delivery, hold the potential to enhance therapeutic efficacy in the CNS. Innovative approaches, such as employing macrophages as carriers, open new avenues for managing conditions like Parkinson's disease.^[31]^ Biodegradable polymers (BDPs) and nano shells offer controlled drug release with reduced toxicity, while liposomes provide a versatile platform for encapsulating diverse therapeutic agents.^[32]^ Dendrimers, with their high branching, improve drug solubility and stability.^[33]^ Nucleic acid‐based nanoparticles (NANPs) are at the vanguard, delivering siRNA and miRNA for gene therapy, heralding a new era in treating genetic and acquired diseases.[^34^, ^35^] In this article, we review the current state‐of‐the‐science in these nanotechnologies for enhancing drug delivery across BBB (Figure 4), and discuss opportunities and challenges for further development and clinical translation. By harnessing these materials, researchers are poised to develop more effective and targeted therapeutic strategies, shedding new light on the treatment of neurological disorders and potentially transforming the landscape of medicine.

## 2 RESEARCH PROGRESS

### 2.1 Structural innovation of nanocarriers

#### 2.1.1 Biodegradable polymers

Biodegradable polymers (BDPs), such as poly(N‐(2‐hydroxypropyl) methacrylamide) (PNPs), are gaining recognition for their potential in enhancing BBB permeability. These polymers can be tailored to improve the solubility, permeability, and bioavailability of therapeutic agents, making them a valuable adjuvants in drug delivery systems.^[36]^ BDPs can serve as drug carriers encapsulating drug molecules within their matrix. This encapsulation protects the drugs from premature degradation in the body and enables controlled release over time.^[37]^ By engineering BDPs with specific properties, targeted drug delivery to the BBB can be achieved.^[38]^ This targeted approach not only increases drug concentration in the brain but also minimizes side effects on peripheral tissues.[^39^, ^40^] The degradation rate of these polymers can be fine‐tuned to match the pharmacokinetic requirements of the drug, optimizing the drug release profile and enhancing therapeutic efficacy.^[41]^

Furthermore, BDPs can enhance the penetration of drug molecules across the BBB by modulating its physical properties or exploiting specific biochemical pathways.^[42]^ Their inherent biocompatibility reduces the immune response triggered by drug carriers, which is particularly beneficial for long‐term treatments.^[35]^ The versatility of BDPs also allows for the integration of multiple functionalities, such as the incorporation of targeting ligands to improve drug specificity or the inclusion of imaging agents for real‐time monitoring of the drug delivery process.^[43]^ With the ongoing advancements in material science and nanotechnology, the application of BDPs in BBB drug delivery is burgeoning. This progress heralds new therapeutic strategies for the treatment of CNS diseases, offering hope for more effective and targeted treatments.[^44^, ^45^]

#### 2.1.2 Magnetic nanoparticles

Nanoparticles can be engineered to carry multiple drugs enabling multimodal therapy. Furthermore, their surfaces can be functionalized with targeting ligands to improve drug specificity.^[46]^ Selecting appropriate materials ensures that these nanoparticles possess good biocompatibility and biodegradability, which is crucial for minimizing potential risks associated with long‐term implantation.^[47]^ The design of responsive carriers, such as pH‐sensitive or enzyme‐sensitive nanoparticles, allows for drug release under specific conditions, thereby further refining the precision of therapy.^[48]^

**FIGURE 5:**
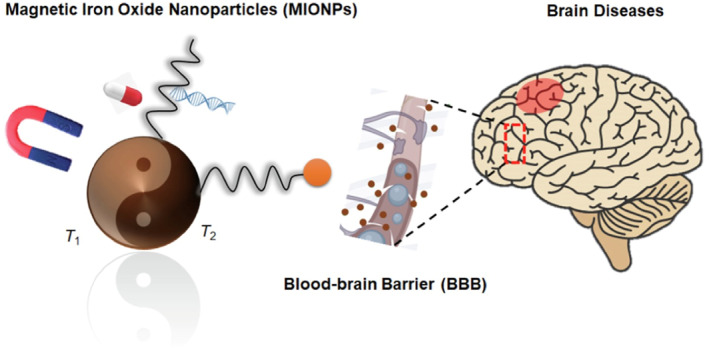
Schematic illustration of magnetic iron oxide nanoparticles (MIONPs) for brain disease imaging and drug delivery.^[49]^ Reproduced with permission. Copyright 2023, Elsevier.

Magnetic nanoparticles are emerging as a promising tool in the realm of BBB permeability,^[47]^ as shown in Figure 5.^[49]^ By exploiting the magnetic properties of these nanoparticles, it is possible to guide drugs with precision to brain lesion areas under the influence of an external magnetic field. This targeted approach enhances both drug penetration through the BBB and therapeutic efficacy.^[50]^ Moreover, the combination of magnetic nanoparticles with ultrasound or magnetic resonance imaging (MRI) technology holds promise for achieving non‐invasive BBB opening, thereby facilitating drug delivery.[^37^, ^51^]

The interaction of magnetic nanoparticles with radiofrequency radiation can release agents that modulate the BBB's integrity, thereby increasing its permeability. This innovative strategy presents new avenues for drug delivery and therapeutic interventions.^[52]^ Additionally, magnetic nanoparticles can generate heat in an alternating magnetic field, a phenomenon known as the magnetothermal effect. This property can be harnessed for the treatment of brain diseases, such as brain tumors, and may also contribute to enhanced BBB permeability.^[53]^

Before transitioning magnetic nanoparticles from preclinical research to clinical applications, extensive evaluation of their safety, efficacy, and potential long‐term effects is imperative.[^46^, ^54^] Successful application of magnetic nanoparticles in enhancing BBB permeability necessitates a multidisciplinary approach involving collaboration across fields such as material science, nanotechnology, biology, and medicine. Such interdisciplinary cooperation is essential for driving innovation and advancing the development of this promising therapeutic modality.^[55]^

#### 2.1.3 Small molecule drug permeability across the BBB

**FIGURE 6:**
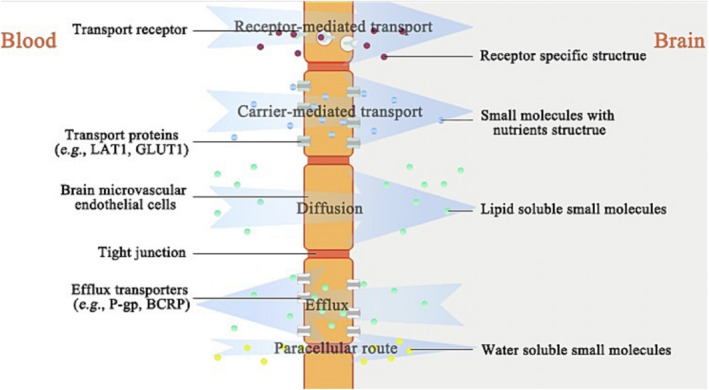
Mechanisms of exchange of small molecules across the BBB.^[57]^ BBB, blood‐brain barrier. Reproduced with permission. Copyright 2021, American Chemical Society.

The BBB is a highly selective semi‐permeable barrier that prevents neurotoxic substances in the blood from entering the CNS. Small molecule drugs, due to their ease of production and transport, as well as the feasibility of structural modification, constitute most clinical treatments for CNS disorders.^[56]^ The mechanism of the exchange of small molecules across the BBB is illustrated in Figure 6.^[57]^ The selectivity of small molecule drugs in crossing the BBB is influenced by several factors including their lipophilicity, molecular size, and the presence of functional groups that can interact with transport proteins or receptors on the BBB.^[58]^ Only a small fraction of lipid‐soluble drugs with a molecular weight lower than 400–600 Da can passively diffuse across the BBB. To enhance the penetration of these drugs, medicinal chemists have attempted various modifications to the molecular structure to improve brain entry or reduce efflux.^[59]^ Common strategies include increasing the lipophilicity of the compound, reducing the number of hydrogen bond donors, removing or replacing negatively charged atoms to lower the topological polar surface area, eliminating basic groups to lower the pKa, and introducing conformational constraints to increase molecular rigidity.^[60]^ Prodrugs designed by linking fragments recognized by transport proteins can be transported into the brain with low diffusion levels, and then released as active molecules through effective enzymatic biotransformation.^[61]^ Therefore, developing effective and safe delivery strategies that can enhance the selectivity of small molecule drugs for the BBB is an urgent issue in the field of drug delivery and neuroscience.

#### 2.1.4 Optical methods in BBB permeability

Utilizing optical methods, particularly femtosecond pulse laser irradiation of molecularly targeted nanoparticles, can safely and reversibly open the BBB and deliver drugs to the brain tissue. The principle involves the generation of a small mechanical wave in the vicinity of the targeted gold nanoparticles by femtosecond laser stimulation, which acts on the tight junctions of the BBB thereby increasing its permeability. This method offers high temporal‐spatial resolution and reversibility and exhibits good safety. Studies have shown that laser‐induced BBB opening does not affect the spontaneous vascular tone of the mouse brain and can effectively deliver typical drugs such as antibody IgG, gene therapy vectors, and drug carriers' liposomes into the brain parenchyma.

#### 2.1.5 Lipid‐based nanoparticles

Lipid‐based nanoparticles (LNPs), encompassing liposomes and solid lipid nanoparticles, constitute a class of delivery vehicles constructed from lipid components.^[62]^ LNPs typically comprise four lipidic components: ionizable lipids, cholesterol, helper lipids, and polyethylene glycol (PEG)‐lipids. These nanoparticles possess the ability to protect nucleic acid payloads from degradation, activate RNA sensing mechanisms, and evade innate immune responses. Additionally, LNPs can introduce nucleic acid payloads into the cytoplasm and function as adjuvants during vaccination.

The application of LNPs in nucleic acid drug delivery is particularly extensive, especially in the realms of mRNA vaccines and cancer therapy. They protect mRNA from degradation and effectively deliver mRNA to cells,^[63]^ thereby facilitating disease treatment. A focal point in LNP research is the development of targeting strategies to selectively deliver payloads to specific cell types, circumventing the issues of inefficacy and toxicity associated with widespread tissue expression.

The advantages of LNPs lie in their biocompatibility and biodegradability which minimize their impact on cell growth and metabolism.^[64]^ Furthermore, the size and surface modification of LNPs can be modulated to optimize their distribution and cellular uptake efficiency in vivo, rendering them an effective nucleic acid delivery system.

#### 2.1.6 Nucleic acid‐based nanoparticles

Nucleic acid‐based nanoparticles represent a cutting‐edge approach in nanomedicine, particularly for gene therapy applications. These nanoparticles can encapsulate genetic material such as siRNA, miRNA, or plasmid DNA,^[65]^ enabling targeted gene silencing or overexpression within the CNS. NANPs can be designed to cross the BBB by exploiting receptor‐mediated transcytosis pathways, which are naturally utilized by viruses and other biomolecules to enter the brain. The precision of NANPs in delivering genetic payloads offers a new dimension in the treatment of genetic and acquired neurological disorders, potentially revolutionizing the field of CNS therapy.

### 2.2 Improvements in targeting strategies

#### 2.2.1 Screening and optimization of targeting ligands

**FIGURE 7:**
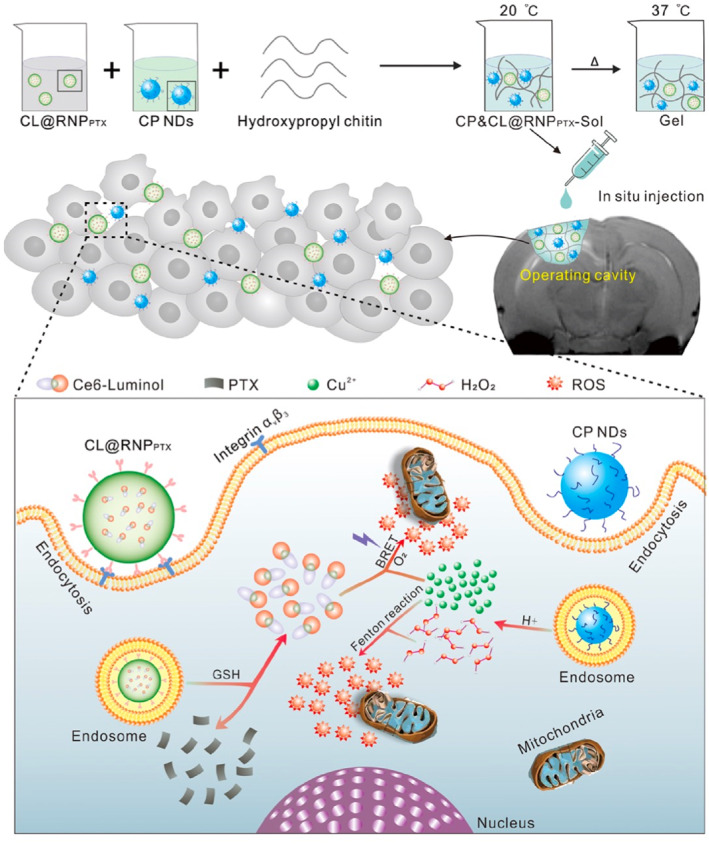
By covalently linking Ce6 with luminol, the Ce6‐luminol (CL) molecule with self‐illuminating potential was synthesized and encapsulated into c(RGDyK) peptide‐modified paclitaxel prodrug nano‐ particles to form a nano system (CL@RNP_PTX_). CP nanodots with pH‐dependent hydroxyl radical (·OH) generation properties were synthesized and embedded into the hydroxypropyl chitin hydrogel 3D framework with CL@RNP_PTX_ to construct a multifunctional nanoparticle‐hydrogel hybrid drug carrier for the treatment of postoperative glioma.^[68]^ Reproduced with permission. Copyright 2022, American Chemical Society.

Targeting ligands are instrumental in enhancing the permeability of the BBB by enabling the specific delivery of therapeutic agents to the CNS.^[66]^ The strategic selection of targeting ligands hinges on identifying molecules with high specificity and affinity for receptors that are overexpressed on the BBB. This targeted approach ensures that therapeutic agents are preferentially delivered to the brain.^[67]^ For example, Hongliang Xin's team designed and embedded a multifunctional nanoparticle into a hydroxypropyl chitin hydrogel to establish a hybrid system (named CP&CL@RNP_PTX_‐Gel) that was designed and fabricated via an interlocking method to realize the combination of photodynamic therapy/chemodynamic therapy/chemotherapy for reinforced, post‐surgical glioma therapy,^[68]^ as shown in Figure 7. These ligands can exploit receptor‐mediated endocytosis pathways to facilitate the transport of drugs across the BBB.^[69]^ This process involves the binding of the ligand to its specific receptor on the BBB endothelial cells, which triggers the internalization of the drug‐ligand complex.[^59^, ^70^] A diverse array of targeting ligands can be utilized, encompassing peptides, antibodies, aptamers, and small molecules, each characterized by distinct properties and binding specificities.^[71]^

The optimization of targeting ligands is crucial for improving the pharmacokinetic profile of drugs, encompassing aspects such as absorption, distribution, metabolism, and excretion,^[72]^ as well as their pharmacological effects at the target site.[^73^, ^74^] It is imperative to ensure that the chosen targeting ligands are biocompatible and do not provoke adverse immune response or exhibit toxicity.^[75]^ The advancement of multifunctional targeting ligands opens the possibility of delivering multiple therapeutic agents simultaneously or integrating imaging agents for real‐time monitoring of the drug delivery process.^[76]^ High‐throughput screening methods are frequently employed to swiftly identify and optimize potential targeting ligands from extensive libraries of candidates.[^77^, ^78^] The ultimate objective of targeting ligand screening and optimization is to facilitate the clinical translation of BBB‐penetrating drugs, ensuring their safety,^[79]^ efficacy, and therapeutic benefits in patients.[^56^, ^80^] This meticulous process is essential for translating preclinical findings into effective clinical therapies, thereby enhancing the treatment of CNS disorders.^[81]^

#### 2.2.2 Development of targeted nanocarriers

**FIGURE 8:**
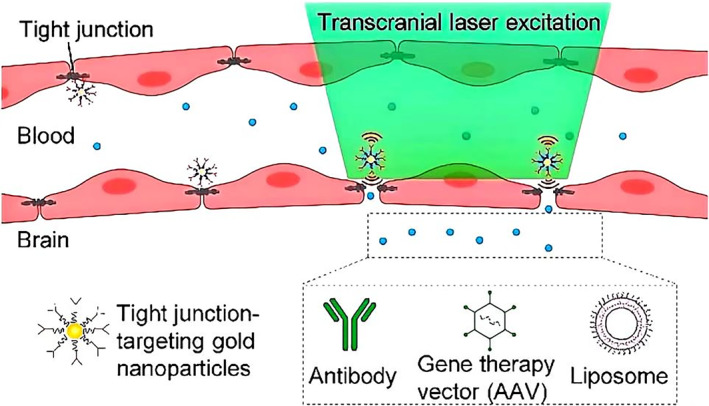
Picosecond stimulation of Tight junction‐targeted nanoparticles reversibly modulate BBB permeability.^[84]^ BBB, blood‐brain barrier. Reproduced with permission. Copyright 2021, American Chemical Society.

The advancement of targeted nanocarriers represents a strategic breakthrough in enhancing BBB permeability, facilitating the targeted delivery of therapeutic agents to the CNS.^[82]^ These nanocarriers are meticulously engineered to incorporate specific targeting ligands that bind with high affinity to receptors on BBB endothelial cells.^[83]^ Zhenpeng Qin's team used picosecond stimulation of Tight junction (TJ‐targeted) nanoparticles to reversibly modulate BBB permeability, as shown in Figure 8.^[84]^ BBB modulation does not lead to significant disruption in the spontaneous vasomotion or the structure of the neurovascular unit. This strategy allows the entry of immunoglobulins and viral gene therapy vectors as well as cargo‐laden liposomes.^[84]^ This design feature not only improves drug delivery across the BBB but also enables controlled release of the payload, ensuring sustained drug levels within the brain and potentially reducing the frequency of administration.^[85]^ By harnessing receptor‐mediated transcytosis pathways, the targeting ligands on these nanocarriers interact with their specific receptors, thereby facilitating the transport of drugs across the BBB.[^83^, ^86^] This approach shields drugs from enzymatic degradation and other clearance mechanisms present at the BBB, significantly increasing their bioavailability in the CNS.[^14^, ^87^]

Some nanocarriers are designed to carry multiple targeting ligands, which can engage different BBB receptors simultaneously, potentially enhancing the overall transport efficiency.[^88^, ^89^] The versatility of these nanocarriers allows them to encapsulate a broad spectrum of therapeutic agents, including small molecules, peptides, nucleic acids, and proteins, offering a flexible approach to treating CNS diseases.[^90^, ^91^] The materials selected for nanocarrier construction are carefully chosen to ensure biocompatibility and minimize the risk of adverse immune responses or toxicity within the body.^[92]^ In certain applications, nanocarriers can be equipped with imaging agents, enabling the visualization of drug distribution and real‐time monitoring of therapeutic efficacy within the brain.[^93^, ^94^]

The development of targeted nanocarriers aligns with the principles of personalized medicine, tailoring treatments to individual patient needs based on genetic or molecular profiles.^[95]^ The ultimate goal is the successful translation of these nanocarriers from the laboratory to clinical practice, offering effective treatments for a variety of CNS disorders.[^96^, ^97^] Driven by advances in nanotechnology, materials science, and a deeper understanding of BBB function, the field of targeted nanocarriers is dynamic and rapidly evolving.^[98]^ These nanocarriers hold immense promise for revolutionizing the treatment of neurological diseases by effectively overcoming the formidable barrier that the BBB presents.^[99]^

#### 2.2.3 Electric field modulation of BBB permeability

Electric field modulation is one of the emerging methods for temporarily and non‐invasively increasing BBB permeability.[^100^, ^101^] Among the approaches are (i) electroporation, the application of short, high‐intensity electric pulses that create transient pores in the endothelial cells of the BBB, thereby increasing permeability.^[102]^ These pores allow the passage of therapeutic molecules (e.g., drugs, nanoparticles) into the brain parenchyma, (ii) electrical stimulation of tight junctions is the application of low‐intensity electric fields that modulate the structure of tight junction proteins (e.g., claudins, occludins and Junctional Adhesion Molecules) in endothelial cells, temporarily reducing the barrier’s integrity,^[103]^ (iii) electrokinetic effects wherein the electric field serve to enhance the transport of charged molecules across the BBB by promoting electrophoresis or electroosmosis, thereby aiding in drug delivery,^[104]^ (iv) tandem effects wherein the electric field is used to achieve synergy in conjunction with other techniques such as chemotheraphy,^[105]^ and possibly focused ultrasound, and microbubbles.^[106]^ Among the advantages, E‐fields are non‐invasive or minimally invasive, their effects are eversible, and when focused, they afford high anatomical precision. Among the challenges, inappropriate E‐fields may damage brain tissue and induce inflammation while its non‐specific permeability changes may allow passage of unwanted agents. Continued research on precise modulation of field intensity, duration, and frequency is necessary for safe and effective BBB opening. Regional heterogeneity of the BBB can affect treatment uniformity.^[107]^ Electric field modulation of BBB permeability is a promising avenue for advancing brain therapeutics, offering new possibilities for overcoming one of the most significant challenges in neuroscience.

### 2.3 Nanotechnologies to enhance imaging‐guided BBB opening

#### 2.3.1 Major imaging techniques for BBB opening guidance

**FIGURE 9:**
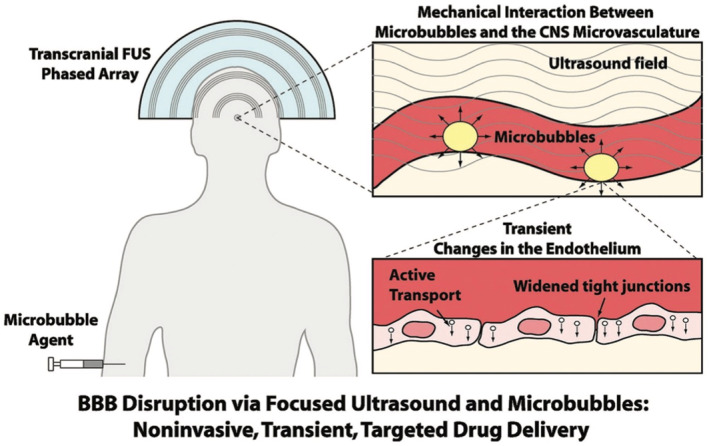
Schematic illustration of ultrasound‐mediated opening of tight junctions.^[7]^ Reproduced with permission. Copyright 2018, Wiley–Blackwell.

Integrating nanomaterials into imaging‐guided BBB opening techniques represents a significant advancement in neurotherapeutic research. In the realm of neurotherapeutics, the quest for effective BBB opening techniques has led to the development of several major imaging‐guided approaches. Doppler color ultrasound stands out as a non‐invasive imaging modality that can modulate the permeability of the BBB.^[108]^ By combining this technique with the use of microbubbles and specific pharmacological agents, a localized effect can be induced, disrupting the tight junctions of the BBB and allowing for its transient permeabilization.^[109]^ What is shown in Figure 9 is that by introducing preformed microbubbles in the presence of a low‐power ultrasound field, the Frank Caruso's group achieved a transient opening of the BBB followed by direct drug passage into the brain parenchyma.^[7]^ The precision of this approach is enhanced through the fine‐tuning of ultrasound intensity and duration, enabling a controlled modulation of BBB permeability.[^109^, ^110^] The application of Doppler color ultrasound for BBB modulation, however, is not without its challenges. Ensuring the uniform distribution of therapeutic agents across the target area and managing the potential side effects of increased BBB permeability are key considerations.^[111]^ Addressing these challenges is crucial for optimizing the safety and efficacy of this technique with the aim of utilizing its potential for targeted drug delivery to the CNS.^[112]^

MRI‐guided BBB opening is another innovative non‐invasive technique that enhances the delivery of therapeutic agents to the brain.[^113^, ^114^] This method typically involves the use of MRI in conjunction with focused ultrasound, where MRI accurately delineates the treatment area, and focused ultrasound applies mechanical effects at precise locations to modulate BBB permeability.^[115]^ The integration of microbubbles under MRI guidance allows anatomical targeting, further enhancing the BBB opening effect.^[116]^

The non‐invasive nature of MRI‐guided BBB opening reduces patient risk and accelerates recovery time, while MRI’s capability for precise imaging allows for personalized treatment plans tailored to individual patient conditions.^[117]^ This technology holds significant potential for the treatment of various brain diseases including brain tumors, neurodegenerative disorders, and CNS infections.^[118]^

In addition to ultrasound and MRI, other imaging modalities such as photoacoustic imaging and positron emission tomography (PET) are being explored to enhance the precision and effectiveness of BBB opening methods. PAI is a non‐invasive imaging technique that combines the high resolution of optical imaging with the deep tissue penetration of ultrasound.^[119]^ When nanomaterials, particularly those with strong optical absorbance, are exposed to pulsed laser light, they generate ultrasound waves that can be detected and used to create high‐resolution images. PAI allows for the real‐time monitoring of BBB opening by visualizing the distribution and concentration of these nanomaterials within the brain. PAI in conjunction with nanomaterials can provide valuable information on the spatial and temporal dynamics of BBB permeability, which is crucial for optimizing drug delivery strategies.^[120]^ PET is a nuclear medicine imaging technique that is highly sensitive for detecting functional changes and molecular processes within the body. In the context of BBB opening, PET can be used to track the biodistribution of radiolabeled nanomaterials and assess their ability to penetrate the BBB.^[121]^ By providing quantitative data on the amount of nanomaterials that have crossed the BBB, PET can help researchers understand the efficacy of various BBB opening strategies and refine their approaches accordingly. The combination of PET with nanomaterials also offers the potential for personalized medicine as it can reveal patient‐specific information about BBB permeability and guide tailored therapeutic interventions.

Incorporating these additional imaging techniques alongside ultrasound and MRI can provide a more comprehensive toolkit for researchers aiming to enhance BBB permeability. The multimodal imaging approach allows for a more detailed understanding of the complex dynamics involved in BBB opening. It can facilitate the development of more targeted and effective therapies for neurological disorders.^[69]^

#### 2.3.2 Nanomaterials for imaging‐guided BBB opening

The integration of nanomaterials into imaging‐guided BBB opening techniques represents a significant advancement in neurotherapeutic research. Nanoparticles, liposomes, and other biomaterials are being explored for their ability to increase drug concentration within the brain when combined with imaging‐guided BBB opening methods.[^117^, ^122^] These nanomaterials can be designed to target specific areas of the brain enhancing the precision of drug delivery and reducing off‐target effects.^[123]^

Polymeric microbubbles represent a class of nano/micro‐sized materials that can significantly enhance BBB permeability under the influence of ultrasound. These microbubbles can be loaded with therapeutic agents enabling targeted drug delivery and real‐time imaging monitoring through the precise manipulation of ultrasound.^[124]^ The cavitation effects induced by the polymeric microbubbles under ultrasound not only facilitate drug transport but also provide a means for real‐time imaging of drug release, which is crucial for enhancing the accuracy and safety of treatments.^[125]^

Furthermore, the application of surface‐enhanced raman scattering (SERS) probes, particularly those constructed from gold nanoparticles, offers a new paradigm for improving imaging sensitivity.^[126]^ After the BBB is opened using ultrasound, SERS probes enable precise imaging of drug distribution and release, significantly enhancing the early diagnostic capabilities for brain diseases.^[127]^

Magnetic nanomaterials also play a pivotal role in drug delivery and imaging. Magnetic nanoparticles can be guided to the target area using an external magnetic field and release their payload post‐BBB opening.^[128]^ The use of MRI allows researchers to monitor the movement and positioning of these nanoparticles in real‐time, demonstrating great potential in targeted therapy for brain tumors.^[129]^

The use of nanomaterials in conjunction with imaging techniques such as MRI‐guided BBB opening allows for a more effective and targeted approach to treating neurological conditions. The versatility of these nanomaterials, coupled with the precision of imaging‐guided techniques, offers a promising avenue for the development of novel treatments for a spectrum of neurological disorders.

### 2.4 Navigating the complexities of BBB modulation in clinic

#### 2.4.1 Assessment of safety and efficacy

The pursuit of effective BBB modulation for the treatment of CNS disorders is a multifaceted endeavor, requiring a delicate balance between innovation and caution.^[130]^ The primary objective in this field is to ensure that any method or substance used for BBB modulation is both safe and efficacious, without causing harm to the patient.^[131]^ This involves meticulous evaluation to avoid damage to neural tissues, minimize inflammation risks, and prevent any long‐term adverse effects, thereby ensuring patient well‐being and safety.^[132]^

Efficacy is equally critical, necessitating a thorough demonstration of a treatment’s ability to cross the BBB and reach the target site within the brain.^[133]^ The therapeutic impact must be scientifically validated through rigorous clinical trials and patient outcomes analysis,^[134]^ which includes continuous monitoring and assessment to detect early adverse effects and evaluate treatment success.

The clinical application of BBB modulation is a dynamic and evolving field, with the potential to revolutionize CNS disorder treatments.^[135]^ Achieving this requires an integrated approach, combining advances in neuroscience, pharmacology, and clinical practice to develop therapies that can effectively target the CNS while maintaining patient safety as the utmost priority.^[130]^

Long‐term follow‐up studies are indispensable for understanding the enduring effects of BBB modulation and monitoring potential delayed adverse effects.^[136]^ These studies are vital for assessing the chronic impact on brain function and structure, identifying delayed reactions early, and ensuring that treatments contribute positively to the long‐term health and well‐being of patients.^[137]^

#### 2.4.2 Current clinical applications of nanomaterials for BBB opening

One of the established clinical applications of nanomaterials in BBB opening is the use of liposomal drugs for the treatment of brain tumors. Liposomal formulations of chemotherapy agents, such as doxorubicin in the form of Doxil®,^[138]^ as described in Table 1, have been used to treat brain metastases due to their ability to preferentially accumulate in tumor tissues. These liposomes can potentially evade the BBB allowing for higher concentrations of the drug to reach the tumor site. In the realm of gene therapy,^[139]^ nanoparticles have been utilized to deliver genetic material across the BBB. For instance, nanoparticles made of polyethyleneimine,^[140]^ as described in Table 1, were explored for their ability to deliver genes to the brain, showing promise in the treatment of genetic disorders like Huntington's disease. Clinical trials are ongoing to assess the safety and efficacy of such approaches.

**TABLE 1:** Summary of nanomaterials for BBB opening in clinical applications.

Drug/nanomaterial name	Mechanism of action	Indications	Clinical status
Liposomal doxorubicin (Doxil®)	Enhanced permeability via liposomal encapsulation	Brain metastases	Approved
Polyethyleneimine (PEI) nanoparticles	Delivery of genetic material across the BBB	Genetic disorders like Huntington's disease	Clinical trials ongoing
Biodegradable polymeric nanoparticles (e.g., PLGA)	Controlled drug release	Various CNS diseases	Phase II clinical trial
Gleevec® (imatinib)	Inhibition of platelet‐derived growth factor receptor, BCR‐ABL, and c‐KIT tyrosine kinases	Chronic myeloid leukemia, gastrointestinal stromal tumors	Approved
Avastin® (bevacizumab)	Humanized monoclonal antibody against vascular endothelial growth factor, inhibiting angiogenesis	Various cancers, including brain tumors	Approved
ADCS‐L01 (Alzheimer’s disease vaccine)	Active immunotherapy targeting amyloid‐beta protein	Alzheimer’s disease	Phase II clinical trial
Prodrug X (hypothetical prodrug)	Hypothetical enzymatic activation in the brain, enhancing BBB permeability	Parkinson’s disease	Phase I clinical trial

Aloïse Mabondzo has presented an innovative in vitro cell culture model that successfully mimics the key characteristics of the BBB and demonstrates its ability to predict the penetration of compounds in the human body using clinical PET radioligand data.^[141]^ The study shows a high correlation between this in vitro model and the permeability of the BBB in humans, offering an efficient and accurate screening tool for drug development. This advancement is instrumental in accelerating the translation of CNS drugs from the lab to clinical applications. Biodegradable polymeric nanoparticles, such as poly (lactic‐co‐glycolic acid) (PLGA), are being used to deliver drugs to the brain in a controlled manner.^[142]^ These nanoparticles can be engineered to release their payload over an extended period, reducing the frequency of administration and potentially enhancing therapeutic outcomes.

In summary, the development and application of BBB‐modulating therapies demand a meticulous, scientifically rigorous process, with a focus on immediate safety and efficacy,^[143]^ as well as long‐term health outcomes.^[144]^ This holistic approach, supported by long‐term data integration and robust clinical trials, is fundamental to advance neurotherapeutics and enhance patient care in the CNS.^[145]^

## 3 RESEARCH PROSPECTS AND CHALLENGES

### 3.1 Artificial intelligence in material design for BBB applications

Artificial Intelligence (AI) has become an indispensable asset in the realm of material design for BBB applications. AI’s capabilities in computational biology have been harnessed to analyze vast datasets, thereby identifying intricate biological patterns that are crucial for understanding BBB interactions.^[146]^ Through the application of machine learning algorithms and extensive datasets, researchers are advancing towards more precise methodologies for assessing the permeability of compounds across the BBB.^[147]^ This approach significantly contributes to the field of CNS drug discovery, particularly in enhancing the understanding of BBB penetration.^[148]^

AI technology is also being employed in the development of micro/nanorobots. These autonomous devices are designed to navigate within the body, breach biological barriers, and accurately locate pathological sites for targeted medication delivery.^[149]^ The inherent complexity of the BBB presents substantial challenges in predicting drug permeability, a critical factor in assessing the efficacy and safety of CNS medications. To address this, researchers are leveraging innovative approaches such as the classification inference of structure‐activity relationships (c‐RASAR) framework, which combines machine learning techniques to enhance predictive capabilities.^[150]^

This integration of AI into the design and analysis of materials for BBB applications represents a significant step forward in the field of neurotherapeutics.^[151]^ By enhancing our ability to predict and understand the interaction of therapeutic agents with the BBB, AI is poised to revolutionize the development and delivery of CNS medications, ultimately improving patient outcomes and treatment options.

### 3.2 Advancements in nanotechnology for CNS disease treatment

The burgeoning field of nanotechnology is paving the way for innovative solutions in the treatment of CNS diseases. Notably, several nanomedicines have either been granted market approval or are currently in clinical trials, showcasing their significant potential in addressing CNS disorders.^[152]^ The advent of functionalized nanomaterials, particularly nanoparticles with the capacity to traverse the BBB, presents a promising avenue for the development of safe and efficacious carriers for neurotherapeutics.^[153]^ These nanoparticles are engineered to enhance BBB permeability through the strategic modulation of their size, shape, charge, and surface ligands.^[129]^ As shown in Table 2.

**TABLE 2:** Comparison of nanomaterials for improving BBB permeability.

Nanomaterial/formula	Mechanism of action	Advantages	Disadvantages
Biodegradable polymers (e.g., PLGA)	Controlled drug release; can be tailored for specific drug delivery	Biocompatibility; tunable degradation rate; potential for targeted drug delivery	Complex synthesis; possible immune response; requires precise control over degradation kinetics
Magnetic nanoparticles	Guided drug delivery using external magnetic fields; can generate heat for therapeutic effects	Precise targeting; potential for multimodal therapy (e.g., drug delivery + hyperthermia)	Potential toxicity from metal ions; requires external magnetic field equipment
Liposomes	Encapsulation of therapeutic agents; protect drugs from degradation	Versatile platform; can encapsulate a variety of drugs; low immunogenicity	Possible aggregation; stability issues in vivo; limited drug loading capacity
Dendrimers	High branching structure improves drug solubility and stability	High drug payload; can be functionalized with multiple targeting groups	Complex synthesis; potential toxicity due to high density of functional groups
Nucleic acid‐based nanoparticles	Gene silencing or overexpression; potential for targeted gene therapy	Precision in gene targeting; potential for treating genetic disorders	Susceptible to degradation by nucleases; challenges in efficient cellular uptake
Lipid‐based nanoparticles (LNPs)	Protection of nucleic acid payloads; evasion of immune responses	Biocompatibility; ability to deliver nucleic acids; potential for targeted delivery	Complexity in formulation; potential for dose‐dependent toxicity

The clinical application of nanotechnology in CNS treatment necessitates a careful consideration of the delivery efficiency of nanoparticles under both intact and compromised BBB states.^[154]^ This variability underscores the need for the development of novel strategies aimed at improving the delivery efficiency of these nanoparticles across diverse pathological conditions.^[155]^ A profound understanding of the mechanisms that underpin the BBB is essential for the successful design of drugs capable of surmounting its biological constraints.[^156^, ^157^] Additionally, a comprehensive grasp of the physicochemical properties of these drugs and their interactions with the BBB is crucial for minimizing adverse side effects and ensuring therapeutic safety.[^14^, ^158^]

Exploration of new nanotechnologies in this context is not merely an academic pursuit but a critical step towards transforming the landscape of CNS disease treatment.^[159]^ By harnessing the power of nanotechnology, researchers are poised to develop more effective therapeutic strategies, ultimately improving patient outcomes and the overall management of CNS diseases.

### 3.3 In‐depth development of interdisciplinary cooperation

The convergence of scientific knowledge and engineering prowess is a catalyst for groundbreaking advancements, particularly in the realms of gene therapy and nanomedicine.^[160]^ These cutting‐edge fields hold the promise of transforming the treatment landscape for CNS diseases.^[161]^ Cross‐disciplinary research teams, comprising experts from diverse domains such as genetics, molecular biology, and materials science, are pivotal in the innovation of novel nanoparticles and drug delivery systems. These systems are designed to selectively target activated glial cells and modulate the BBB, thereby enhancing therapeutic efficacy.^[162]^

Striking a balance between maintaining scientific rigor and fostering interdisciplinary collaboration is essential. This endeavor necessitates a nuanced approach to integrating diverse academic traditions and evaluation standards.^[163]^ The complexity of merging fields such as engineering, economics, business, social sciences, and psychology requires a deep respect for the distinct methodologies and perspectives each discipline brings to the table.^[164]^ This integration is not merely an academic exercise but a strategic imperative for driving innovation and addressing the multifaceted challenges inherent in CNS disease research and treatment.

By embracing the richness of interdisciplinary cooperation, the scientific community can harness a broader spectrum of insights and expertise,^[165]^ ultimately accelerating the pace of discovery and the development of more effective therapies for CNS diseases.^[166]^ This collaborative approach is crucial for navigating the intricate interplay of biological,^[167]^ technological, and social factors that influence the progression and management of these conditions.

### 3.4 Challenges and discussion

The transition of nanotechnologies from the lab to the clinic is a complex process that involves several significant challenges. A primary concern is the biocompatibility and potential toxicity of nanomaterials. Ensuring that these materials can interact with the BBB without inciting adverse immune responses is crucial, and this requires long‐term safety assessments.^[168]^

Manufacturing nanomaterials at a scale suitable for clinical use (cGMP) while maintaining consistent physicochemical properties is another significant hurdle.^[169]^ The consistency of particle characteristics is essential for the reproducibility and reliability of therapeutic outcomes. The successful scale‐up of biodegradable nanoparticles for controlled drug release requires sophisticated processes that can maintain the delicate balance of particle size and surface properties, which are known to influence biodistribution and cellular uptake.

The specificity of nanomaterial targeting to the BBB is also a complex issue. While there have been advances in the development of targeting ligands, ensuring high affinity and specificity for BBB receptors remains a challenge.[^170^, ^171^] This is exemplified in the ongoing clinical trials for nanoparticle‐mediated gene therapy, where the ability to selectively deliver genetic material across the BBB is critical for treating diseases like Huntington's disease.

Patient heterogeneity further complicates the clinical application of nanomaterial‐based therapies. Variability in BBB integrity and function across patient populations necessitates personalized medicine approaches.^[172]^ This is particularly relevant in the context of brain tumors, where the heterogeneity of the tumor microenvironment can significantly impact the efficacy of nanomaterial‐based drug delivery. The anatomical regional heterogeneity of the BBB (e.g., the density of capillaries), expression levels of tight junction proteins, region‐specific expression of *efflux transporters* (e.g., P‐glycoprotein, BCRP) and *influx transporters* (e.g., GLUT1 for glucose) implies that a single platform technology may not be a panacea for all CNS pathologies.

Clinical application of liposomal doxorubicin for brain tumors exemplifies the multifaceted challenges in translating nanotechnologies across the BBB. One of the primary concerns is ensuring the biocompatibility and safety of these nanomaterials as they must interact with the BBB without provoking adverse immune responses.^[173]^ This necessitates extensive long‐term safety assessments to evaluate potential toxicity. Additionally, the manufacturing process poses significant hurdles. Variability in particle size, charge, and encapsulation efficiency can directly influence drug biodistribution and therapeutic efficacy.

To overcome these obstacles, a multidisciplinary approach that combines advanced materials science, innovative targeting strategies, and close collaboration with regulatory bodies is necessary. This will facilitate the development of more effective and targeted therapeutic strategies that can successfully navigate the complexities of the BBB and improve clinical outcomes for patients with brain tumors.

## 4 CONCLUSION

The saga of BBB research is a testament to the relentless pursuit of discovery and innovation. Since Paul Ehrlich first unveiled its enigmatic nature in the 1880s, and Thomas Reese and Morris Karnovsky further demystified its structure by visualizing endothelial cells in 1967, our understanding of the BBB has evolved significantly. Over time, scientists have delved into the physiological structure and function of the BBB, pioneering techniques such as electroporation to enhance its permeability and pave the way for novel drug delivery methods.

In recent years, the advent of nanotechnology and ultrasound techniques has heralded new horizons for targeted therapy. Material science has emerged as a pivotal force in enhancing BBB permeability, thereby expanding the frontiers of drug delivery to the CNS.^[174]^ Innovations in this field hold the promise of a transformative impact on CNS drug delivery by modulating BBB permeability.^[175]^ Advances in materials for culturing brain endothelial cells, in vitro BBB permeability assessment methods, permeability‐enhancing strategies, and the development of cargo‐loaded nanoparticles have collectively propelled researchers toward overcoming the formidable challenges of the BBB.[^176^, ^177^] Safety research and clinical applications are also gaining momentum. Moreover, the integration of biological materials and AI in material design is not only propelling clinical trials but also offering innovative pathways for material optimization.

As we look to the future, the evolution of nanotechnology and the intensification of interdisciplinary cooperation will undoubtedly present new challenges and opportunities in BBB research. These developments are set to further catalyze the innovation of drug delivery and treatment strategies, ultimately contributing more significantly to human health. Interdisciplinary cooperation is the conduit through which knowledge from varied disciplines is integrated, fostering a holistic comprehension of intricate issues and phenomena. By nurturing collaboration among experts from diverse fields, interdisciplinary efforts facilitate the exchange of ideas, expertise, and resources, culminating in groundbreaking solutions and progress. This collaborative ethos is the cornerstone of our ongoing quest to unravel the complexities of the BBB and harness its potential for the betterment of human health.

## CONFLICT OF INTEREST STATEMENT

Prof. Guiseppi‐Elie is the founder, president, and scientific director of ABTECH Scientific, Inc., a manufacturer of microfabricated electrodes, devices and systems used in biomedical diagnostics and the measurement of physiological data.

## Data Availability

Data sharing is not applicable.
